# The effect of head and neck basal cell carcinoma on body image, quality of life, and Skin Cancer Quality of Life Impact Tool – Experience of a tertiary dermatology clinic: A cross-sectional study

**DOI:** 10.1097/MD.0000000000043599

**Published:** 2025-08-01

**Authors:** Ozan Yildirim, Nazli Caf, Mustafa Tümtürk, Zafer Türkoğlu

**Affiliations:** a Department of Dermatology and Venerology, Başakşehir Çam and Sakura City Hospital, Istanbul, Türkiye; b Faculty of Medicine, Department of Dermatology and Venerology, Istanbul Atlas University, Istanbul, Türkiye.

**Keywords:** Basal cell carcinoma, Body Image Scale, dermatological quality of life, Skin Cancer Quality of Life Impact

## Abstract

The aim of this study was to examine the impact of head and neck localized basal cell carcinoma (BCC) on body image, dermatological quality of life, and skin cancer-related quality of life. A total of 119 patients were enrolled. Patients diagnosed with BCC were asked to complete the Dermatology Life Quality Index (DLQI), Body Image Scale (BIS), and Skin Cancer Quality of Life Impact Tool (SCQOLIT). Additionally, demographic information, tumor type, localization, size, and duration of the tumors were recorded. Descriptive statistics, including mean, standard deviation, median, minimum, maximum, frequency, and ratio values, were calculated. Among the 119 patients, 45% were female, and 55% were male. SCQOLIT scores for BCC ranged from 0 to 11, with a mean score of 5.56 ± 2.14. The BIS scores ranged from 1 to 7, with a mean score of 3.16 ± 1.27. The DLQI scores ranged from 0 to 4, with a mean score of 1.74 ± 1.01. The average score of the SCQOLIT was 5.56 ± 2.14, indicating that the quality of life of BCC patients was moderately affected in the past week. The average BIS score was 3.16 ± 1.27, suggesting that patients experienced a minimal impact on their body perception. The average DLQI score was 1.74 ± 1.01, revealing that BCC had a minimal impact on patients’ dermatological quality of life.

## 1. Introduction

Basal cell carcinoma (BCC) is the most common cutaneous malignancy arising in individuals over the age of 50. It is more frequently encountered in people with fair skin types and typically exhibits slow-growing behavior, with metastasis being exceedingly rare. While BCC predominantly arises in the head and neck region, it may develop in any area of the body.^[1,2]^

Dermatological disorders profoundly affect appearance, daily life, psychosocial well-being, and relationships. Thus, specialized tools or questionnaires are essential to evaluate their impact on patients’ lives, psychosocial status, and treatment perceptions.^[3]^

The Dermatology Life Quality Index (DLQI) is the most widely used tool for assessing quality of life in dermatological diseases. As the first dermatology-specific quality-of-life assessment instrument, the DLQI has been translated into numerous languages and continues to be utilized globally today. The DLQI is a simple 10-item questionnaire that assesses the impact of dermatological symptoms on daily activities, relationships, and work over the past week. It helps physicians understand patients’ disease-related difficulties but offers limited insight into emotional status. Scores range up to 30, with higher scores reflecting greater quality-of-life impairment.^[4]^

The Skin Cancer Quality of Life Impact Tool (SCQOLIT) was specifically developed to evaluate the quality of life in patients with melanoma and nonmelanoma skin cancers (NMSC). The SCQOLIT is a 10-item questionnaire designed to measure the quality of life over the past 7 days in patients diagnosed with nonmetastatic malignant melanoma (MM) and NMSC.^[5]^ The SCQOLIT scale has no cutoff value, with higher values indicating greater impact.^[5,6]^ Several studies in the literature have compared SCQOLIT scores between patients with MM and those with NMSC, with findings consistently indicating higher SCQOLIT scores among patients diagnosed with MM.^[5–7]^

Body image refers to an individual’s perception of their own body, including its appearance, limbs, and organs, as shaped by their mind. This perception can vary significantly depending on factors such as age, social environment, educational background, culture, and personality traits. The head and neck regions, being highly visible and directly exposed, play a critical role in influencing a person’s social interactions, educational or professional pursuits, and even their sexual life. Given the prominence of these regions, it is reasonable to assume that they can significantly impact an individual’s body image. Recent studies have displayed that patients having tumors on their head and neck are at increased risk of body image dissatisfaction due to the visible disfigurement that may occur from their illness and/or its treatment. This dissatisfaction is associated with difficulty coping, anxiety, and/or depression, further effecting their quality of life. Besides, the psychosocial role of body image in the quality of life of head and neck cancer patients has been widely reviewed, addressing the need for comprehensive care approaches for the solution of these concerns.^[8,9]^ The Body Image Scale (BIS) is a psychometric tool developed to assess the psychological effects of diseases and treatments on patients’ perception of their physical appearance.^[10–12]^ It consists of 10 questions related to external appearance. Although there is currently no universally accepted cutoff value for BIS, there are authors in the literature who accept a cutoff of 8 or 10.^[11,13]^ We accepted a cutoff of 8, aiming for the lower threshold would be more easily captured in patients with a more subtle body image disorder.

The SCQOLIT was specifically developed for NMSC^[14]^ but still remains underexplored in the literature. Similarly, although the BIS has been applied in studies involving head and neck cancers,^[8,9]^ it has not been studied exclusively in the context of BCC. These gaps in existing literature assisted our decision to utilize these 2 scales, aiming to address this particular deficiency through our study.

Given these overlooked conditions mentioned above, we aimed to design a study addressing this gap by focusing on head and neck BCC and its impact on body image and quality of life. The primary aim of this study is to investigate the impact of tumors on body image and quality of life in patients diagnosed with BCC located in the head and neck region, utilizing the BIS, DLQI, and SCQOLIT.

## 2. Materials and methods

This study was conducted as a prospective, cross-sectional observational survey at the Dermatology Outpatient Clinic of T.C. Health Sciences University, Başakşehir Çam and Sakura City Hospital between April 1, 2023 and January 1, 2024. Ethical approval for the study was obtained from the Ethics Committee of the T.C. Health Sciences University, Başakşehir Çam and Sakura City Hospital (Approval Number: 2023.03.122, Issue Number: KAEK/2023.03.122).

Patients aged 45 to 65 years who presented consecutively to the dermatology outpatient clinic and were diagnosed with BCC based on clinical, dermoscopic, and histopathological evaluations were considered for inclusion. All patients deemed eligible according to the study criteria were recruited consecutively after providing written informed consent. The study was conducted in accordance with the Declaration of Helsinki.

Data on patients’ demographic characteristics, educational background, smoking and alcohol habits, sunscreen usage, history of severe burns, and personal or family history of skin cancer were recorded. The study form also documented the patient’s Fitzpatrick skin type, tumor location and diameter, number, and duration of lesions. In addition, the dermoscopic findings of the patients were recorded in the study form.

In the subsequent stage, patients were asked to complete the DLQI, BIS, and SCQOLIT. The scores obtained from these questionnaires were recorded in the patient files by the physicians involved in the study.

Patients who were illiterate or had a diagnosis of anxiety or depressive disorders, a prior diagnosis of personality or bipolar disorder, eating disorders, or those using psychiatric medications were excluded. Patients over the age of 65 were also excluded from this study due to the potential for cognitive impairment.

The Statistical Package for Social Sciences version 26.0 software program (IBM Corp., Armonk) was used for the analysis of the study data. Descriptive statistics, including mean, standard deviation, median, minimum, maximum, frequency, and percentage values, were employed to summarize the data. The Pearson chi-square test was applied to assess relationships between categorical variables. The Shapiro–Wilk test was utilized to determine whether the data conformed to a normal distribution. For comparisons between independent groups, the Mann–Whitney *U* test and the Kruskal–Wallis *H* test were performed. A *P*-value of < .05 was considered statistically significant.

## 3. Results

### 3.1. Demographic data

A total of 119 patients having head and neck-localized BCC were included (45% female, 55% male) with a mean age of 57.08 ± 5.28 years (range: 45–65) (Table 1).

**Table 1:** Participants’ demographic data – frequencies.

	Frequency	Percentage (%)
Gender		
Female	54	45.4
Male	65	54.6
Age (yr)		
45–50	14	11.8
51–55	29	24.4
56–60	39	32.8
61–65	37	31.1
Educational status		
Illiterate	22	18.5
Primary school	64	53.8
High school	27	22.7
University	6	5.0
Skin type (Fitzpatrick)		
1	16	13.4
2	49	41.2
3	47	39.5
4	7	5.9
Smoking habit		
No	77	64.7
Yes	42	35.3
Alcohol use		
No	98	82.4
Yes	21	17.6
Habit of using sunscreen		
No	112	94.1
Yes	7	5.9
Use sunscreen only during summer		
No	0	0
Yes	7	100.0
Comorbidities		
None	55	46.2
Hypertension	49	41.2
Diabetes mellitus	32	26.9
Congestive heart failure	14	11.9
Rheumatoid Arthritis	2	1.7
Hypothyroidism	2	1.7
Chronic renal failure	2	1.7
Addison disease	1	0.8
Chronic obstructive pulmonary disease	1	0.8
Family history of skin cancer		
No	104	87.4
Yes	15	12.6

The mean BMI was 28.30 ± 1.79 kg/m^²^. Regarding education, 54% completed primary school, 23% high school, 5% university, and 18% had no formal education. Occupationally, 29% were housewives, 23% civil servants, 15% retirees, 13% tradespeople, and 8% each were farmers and laborers (Tables 1 and 2).

**Table 2:** Demographic characteristics of participants.

	n	Mean	SD	Min	Max
Age	119	57.08	5.28	45	65
Height (cm)	119	169.9	8.03	157	186
Weight (kg)	119	81.97	9.43	65	112
BMI (kg/m²)	119	28.30	1.79	24.2	35.6

max = highest score received, min = lowest score received, SD = standard deviation.

Fitzpatrick skin types were as follows: type I (13%), type II (41%), type III (39%), and type IV (6%) (Table 1).

In the study, 35% of participants were smokers, and 18% consumed alcohol (Table 1). A total of 94% of patients reported no regular use of sunscreen, while the remaining 6% used it exclusively during the summer months.

None of the participants reported a history of severe sunburn during childhood or adulthood. Comorbidities were identified in 46% of the patients, with 41% having hypertension, 27% type II diabetes mellitus, and 12% hypothyroidism (Table 1).

A family history of skin cancer was reported by 13% of the participants (Table 1).

The age of BCC onset ranged from 41 to 65 years (mean: 54.65 ± 5.82), with the majority between 51 and 60 years (Table 1).

### 3.2. Clinical features

A solitary BCC was observed in 87% of participants, while one patient had 10 lesions (Table 3).

**Table 3:** BCC characteristics of the patients.

	Frequency	Percentage (%)
Number of BCCs		
1	103	86.6
2	12	10.1
3	3	2.5
10	1	0.8
BCC type		
Nodular	55	46.2
Nodular and pigmented	4	3.4
Superficial	10	8.4
Morpheiform/infiltrative	3	2.5
Micronodular	4	3.4
Pigmented	34	28.6
Basosquamous	5	4.2
Adenoid	4	3.4
BCC localization		
Nose	57	47.9
Malar	14	11.8
Neck	14	11.8
Periorbital	12	10.1
Temporal	10	8.4
Frontal	9	7.6
Jaw	4	3.4
Ear	4	3.4
Scalp	3	2.5
BCC dermoscopic findings		
Arborizing vessels	108	90.8
Ulceration	72	60.5
Short fine telangiectasias	71	59.7
Blue gray globules	50	42.0
Leaf-like structures	13	10.9
Large blue/gray oval structures	11	9.0
Spoke wheel-like structures	10	8.4
Concentric structures	9	7.6

BCCs were most commonly located on the nose (48%), followed by the malar region and neck (12% each), periorbital (10%), temporal and frontal areas (8% each), chin and ear (3% each), and scalp (2%) (Table 3).

Lesion duration ranged from 2 to 120 months (mean: 27.86 ± 19.98 months). Tumor diameters ranged from 0.5 to 5 mm (mean: 1.59 ± 0.81 mm).

Dermoscopic findings included: branching vessels (91%), ulceration and fine telangiectasias (60% each), blue-gray globules (42%), leaf-like structures (11%), blue-gray ovoid nests (9%), and spoke-wheel/concentric structures (8%) (Table 3).

### 3.3. Scales

SCQOLIT scores ranged from 0 to 11 (mean: 5.56 ± 2.14), BIS from 1 to 7 (mean: 3.16 ± 1.27), and DLQI from 0 to 4 (mean: 1.74 ± 1.01) (Table 4).

**Table 4:** Descriptive statistics of participants’ scale scores.

Scales	n	Mean ± SD	*M* (Min–Max)	*P*
Skin Cancer-related Quality of Life	119	5.56 ± 2.14	6 (0–11)	.031
Body Image Scale	119	3.16 ± 1.27	3 (1–7)	.000
Dermatological Life Quality Index	119	1.74 ± 1.01	2 (0–4)	.000

*M* = median, *P* = Shapiro–Wilk test.

The Shapiro–Wilk test was conducted to assess the normality of the scale distributions, revealing a deviation from normality (*P* < .05).

No significant gender differences were found for any scale (Mann–Whitney *U* test, *P* > .05) (Table 5).

**Table 5:** Comparison of participants’ scale scores according to demographic information.

Scales	Group	n	Mean ± SD	*M* (Min–Max)	*P*
Gender					
Skin Cancer-related Quality of Life Index	Female	54	5.87 ± 1.99	6 (0–10)	.119†
	Male	65	5.31 ± 2.23	5 (1–11)	
Body Image Scale	Female	54	3.30 ± 1.31	3 (1–7)	.423†
	Male	65	3.05 ± 1.23	3 (1–6)	
Dermatological Life Quality Index	Female	54	1.72 ± 1.11	2 (0–4)	.893†
	Male	65	1.75 ± 0.92	2 (0–4)	
Age					
Skin Cancer-related Quality of Life Index	45–50	14	6.93 ± 2.27	6.5 (4–11)	.106‡
	51–55	29	5.17 ± 1.91	5 (2–9)	
	56–60	39	5.69 ± 2.12	6 (0–9)	
	61–65	37	5.22 ± 2.14	5 (1–9)	
Body Image Scale	45–50	14	4.0 ± 1.41	4 (2–7)	.099‡
	51–55	29	3.03 ± 1.18	3 (1–6)	
	56–60	39	2.95 ± 1.39	3 (1–6)	
	61–65	37	3.16 ± 1.04	3 (1–5)	
Dermatological Life Quality Index	45–50	14	2.0 ± 1.18	2 (0–4)	.563‡
	51–55	29	1.86 ± 0.83	2 (0–3)	
	56–60	39	1.64 ± 0.96	2 (0–4)	
	61–65	37	1.65 ± 1.11	2 (0–4)	
Smoking habit					
Skin Cancer-related Quality of Life Index	No	77	5.38 ± 2.18	6 (0–10)	.256†
	Yes	42	5.91 ± 2.03	6 (2–11)	
Body Image Scale	No	77	3.01 ± 1.30	3 (1–7)	.073†
	Yes	42	3.43 ± 1.17	3 (1–6)	
Dermatological Life Quality Index	No	77	1.90 ± 0.94	2 (0–4)	.021†*
	Yes	42	1.45 ± 1.06	1 (0–4)	
Alcohol use					
Skin Cancer-related Quality of Life Index	No	98	5.54 ± 2.40	6 (0–11)	.819†
	Yes	21	5.67 ± 1.62	6 (3–9)	
Body Image Scale	No	98	3.03 ± 1.27	3 (1–7)	.014†,*
	Yes	21	3.76 ± 1.09	4 (2–6)	
Dermatological Life Quality Index	No	98	1.83 ± 0.97	2 (0–4)	.061†
	Yes	21	1.33 ± 1.06	1 (0–3)	
Habit of using sunscreen					
Skin Cancer-related Quality of Life Index	No	112	5.56 ± 2.09	6 (1–11)	.715†
	Yes	7	5.57 ± 2.99	6 (0–9)	
Body Image Scale	No	112	3.17 ± 1.29	3 (1–7)	.776†
	Yes	7	3.00 ± 0.82	3 (2–4)	
Dermatological Life Quality Index	No	112	1.70 ± 0.98	2 (0–4)	.145†
	Yes	7	2.28 ± 1.25	2 (0–4)	
Skin type (Fitzpatrick)					
Skin Cancer-related Quality of Life Index	1	16	5.62 ± 1.63	6 (2–9)	.900‡
	2	49	5.41 ± 2.25	5 (0–10)	
	3	47	5.64 ± 2.11	6 (1–11)	
	4	7	6.00 ± 2.83	8 (3–9)	
Body Image Scale	1	16	3.19 ± 1.51	4 (2–7)	.907‡
	2	49	3.18 ± 1.38	3 (1–7)	
	3	47	3.19 ± 1.06	3 (1–6)	
	4	7	2.71 ± 1.38	3 (1–4)	
Dermatological Life Quality Index	1	16	1.63 ± 1.15	1.5 (0–3)	.925‡
	2	49	1.79 ± 0.99	2 (0–4)	
	3	47	1.74 ± 0.99	2 (0–4)	
	4	7	1.57 ± 0.98	2 (0–3)	
Educational status					
Skin Cancer-related Quality of Life Index	Illiterate	22	5.82 ± 2.24	6 (2–9)	.854‡
	Primary school	64	5.42 ± 1.93	6 (2–9)	
	High school	27	5.67 ± 2.63	6 (0–11)	
	University	6	5.67 ± 1.75	5 (4–9)	
Body Image Scale	Illiterate	22	3.24 ± 0.97	3 (1–5)	.877‡
	Primary school	64	3.09 ± 1.32	3 (1–6)	
	High school	27	3.22 ± 1.48	3 (1–7)	
	University	6	3.33 ± 0.82	3.5 (2–4)	
Dermatological Life Quality Index	Illiterate	22	1.72 ± 1.16	2 (0–4)	.069‡
	Primary school	64	1.56 ± 0.99	2 (0–4)	
	High school	27	2.07 ± 0.87	2 (0–3)	
	University	6	2.17 ± 0.75	2 (1–3)	

*M* = median, max = highest score received, min = lowest score received, SD = standard deviation.

† Mann–Whitney *U* test.

‡ Kruskal–Wallis *H* test.

* *P* < .05; there is a statistically significant difference between the groups.

No age-related differences were observed in SCQOLIT, BIS, or DLQI scores (Kruskal–Wallis, *P* > .05) (Table 5).

The Mann–Whitney *U* test was used to assess differences in scale scores based on smoking status. No statistically significant differences were found in the SCQOLIT or BIS scores between smokers and nonsmokers (P > .05). However, a statistically significant difference was observed in DLQI scores (*P* = .021), with smokers having higher mean scores (Table 5).

The Mann–Whitney *U* test was performed to evaluate differences in scale scores based on alcohol use. No statistically significant differences were found in the SCQOLIT or DLQI scores between alcohol users and nonusers (P > .05). However, a statistically significant difference was observed in BIS scores (*P* = .014), with alcohol users having higher mean scores (Table 5).

### 3.4. Statistical relationships between scales

SCQOLIT and BIS showed a moderate positive correlation (*R* = 0.300), but no correlation was found between SCQOLIT and DLQI (P > .05) according to the Spearman correlation test (Table 6).

**Table 6:** Relationship between participants’ scale scores.

Variables		1	2
1	Skin Cancer-related Quality of Life Index	–	–
2	Body Image Scale	.300*	–
3	Dermatological Life Quality Index	.137	−.079

* P < 0.05.

Educational level did not affect SCQOLIT, BIS, or DLQI scores according to Kruskal–Wallis *H* test (P > .05).

BCC localization showed no significant impact on any of the scales according to the Mann–Whitney *U* test (P > .05) (Table 7).

**Table 7:** Comparison of scale scores of participants according to BCC type.

Scales	BCC type and localization	n	Mean ± SD	*M* (Min–Max)	*P*
Skin Cancer-related Quality of Life Index	Nodular	55	3.22 ± 1.29	3 (1–7)	.625†
	Nodular and pigmented	4	3.25 ± 1.26	3 (2–5)	
	Superficial	10	3.10 ± 1.45	3 (1–6)	
	Morpheiform/infiltrative	3	3.33 ± 2.31	2 (2–6)	
	Micronodular	4	3.0 ± 1.41	2 (2–5)	
	Pigmented	34	3.09 ± 1.54	3 (1–5)	
	Basosquamous	5	2.8 ± 1.30	2 (2–5)	
	Adenoid	4	3.5 ± 0.58	3.5 (3–4)	
Body Image Scale	Nodular	55	5.78 ± 2.15	6 (0–10)	.954†
	Nodular and pigmented	4	5.25 ± 2.63	4.5 (3–9)	
	Superficial	10	5.5 ± 3.03	6 (1–11)	
	Morpheiform/infiltrative	3	5.67 ± 1.53	6 (4–7)	
	Micronodular	4	6.0 ± 1.63	6 (4–8)	
	Pigmented	34	5.38 ± 2.00	5.5 (2–9)	
	Basosquamous	5	4.0 ± 1.22	4 (2–5)	
	Adenoid	4	6.0 ± 2.45	6 (3–9)	
Dermatological Life Quality Index	Nodular	55	1.55 ± 1.05	2 (0–3)	.617†
	Nodular and pigmented	4	2.0 ± 0.82	2 (1–3)	
	Superficial	10	2.10 ± 0.87	2 (1–3)	
	Morpheiform/infiltrative	3	2.33 ± 1.15	3 (1–3)	
	Micronodular	4	1.5 ± 1.29	1.5 (0–3)	
	Pigmented	34	1.82 ± 0.97	2 (0–4)	
	Basosquamous	5	1.8 ± 0.45	2 (1–2)	
	Adenoid	4	2.25 ± 1.26	2 (1–4)	
Skin Cancer-related Quality of Life Index	Nose	57	2.98 ± 1.38	3 (1–7)	.293*
	Malar	14	3.5 ± 1.34	3 (2–6)	.780*
	Neck	14	3.43 ± 1.45	3.5 (1–6)	.891*
	Periorbital	12	6.17 ± 1.75	6 (4–9)	.285*
	Temporal	10	3.2 ± 0.79	3 (2–4)	.552*
	Forehead	9	3.0 ± 1.0	3 (2–4)	.576*
	Chin	4	3.5 ± 1.29	3.5 (2–5)	.571*
	Ear	4	3.5 ± 1.29	3.5 (2–5)	.522*
	Scalp	3	2.67 ± 1.53	3 (1–4)	.060*
Body Image Scale	Nose	57	5.32 ± 2.24	5 (0–10)	.106*
	Malar	14	5.43 ± 1.74	5.5 (3–9)	.369*
	Neck	14	5.79 ± 2.58	5.5 (2–11)	.355*
	Periorbital	12	3.58 ± 0.99	4 (2–5)	.153*
	Temporal	10	5.9 ± 2.23	6 (2–9)	.783*
	Forehead	9	5.0 ± 2.60	6 (1–8)	.745*
	Chin	4	5.0 ± 2.45	6 (3–8)	.559*
	Ear	4	5.0 ± 2.95	4.5 (2–9)	.559*
	Scalp	3	3.33 ± 1.53	3 (2–5)	.635*
Dermatological Life Quality Index	Nose	57	1.87 ± 1.82	2 (0–4)	.475*
	Malar	14	1.5 ± 1.34	1 (0–4)	.328*
	Neck	14	1.43 ± 1.16	1.5 (0–3)	.287*
	Periorbital	12	1.58 ± 0.99	2 (0–3)	.615*
	Temporal	10	1.7 ± 1.16	1.5 (0–4)	.711*
	Forehead	9	2.11 ± 1.17	2 (1–4)	.372*
	Chin	4	2.0 ± 0.82	2 (1–3)	.595*
	Ear	4	1.75 ± 0.5	2 (1–2)	.994*
	Scalp	3	1.33 ± 0.58	1 (1–2)	.430*

*M* = Median, max = highest score received, min = lowest score received, SD = standard deviation.

* Mann–Whitney *U* test.

† Kruskal–Wallis *H* test.

*P* < .05; there is a statistically significant difference between the groups.

The Spearman correlation test was conducted to examine the relationship between scale scores and BCC diameter among the participants. No statistically significant correlations were found between BCC diameter and SCQOLIT, BIS, or DLQI (P > .05 for all) (Table 8).

**Table 8:** Relationship between participants’ scale scores and BCC diameter.

Variables	BCC diameter
Skin Cancer-related Quality of Life Index	−.052
Body Image Scale	.130
Dermatological Life Quality Index	−.108

P < 0.05.

While no significant differences were found in SCQOLIT or DLQI across occupations (P > .05) but BIS scores varied (*P* = .034), with tradespeople scoring highest and farmers lowest according to the Kruskal–Wallis *H* test.

Age of BCC onset showed no significant correlation with any of the used scales according to the Spearman correlation test (P > .05 for all).

The Spearman correlation test was performed to assess the relationship between participants’ scale scores and the number of BCC lesions. A negative, weak correlation was observed between SCQOLIT and the number of BCCs (*R* = −0.222), suggesting that higher scores on SCQOLIT were associated with fewer BCCs. No statistically significant relationships were found between the number of BCCs and the BIS or the DLQI (P > .05 for both) (Table 9).

**Table 9:** Relationship between participants’ scale scores and number of BCC lesions.

Variables	Number of BCC lesions
Skin Cancer-related Quality of Life Index	−.222*
Body Image Scale	−.018
Dermatological Life Quality Index	−.007

* P < 0.05.

The Spearman correlation test was conducted to evaluate the relationship between participants’ scale scores and lesion duration. No statistically significant correlations were found between lesion duration and SCQOLIT, BIS, or DLQI scores (P > .05 for all). Additionally, no statistically significant relationship was observed between lesion duration and the number of BCCs (*P* > .05).

## 4. Discussion

BCC is the most common skin tumor observed in individuals over the age of 50. The age of onset for BCC varies across studies and is known to be influenced by geographical differences. While BCC is typically seen in individuals aged 50 to 80 years, numerous studies have reported its emergence in younger age groups.^[15]^ Given the increasing average age of populations worldwide, it is evident that BCC is becoming, and will continue to be, a significant public health concern.

The skin, as the largest organ of the body and one that is externally visible, plays a crucial role in body image and social interactions. While reflecting overall health, our skin also influences self-confidence, self-perception, quality of life, and how we are perceived by others.^[3]^

In dermatology, various questionnaires and scales are available for different purposes. Among these, DLQI is the most commonly used. The DLQI consists of 10 simple, concise, and clear questions, forming a self-assessment tool for patients evaluating over the past week.^[16]^ Higher scores are associated with impaired quality of life.^[16]^ In our study, BCC patients had a mean DLQI score of 1.74, indicating minimal impairment, consistent with prior findings that BCC generally has a limited effect on patients’ dermatological quality of life. The BIS is a self-assessment questionnaire comprising 10 items related to an individual’s perception of their external appearance with no exact established cutoff value. Higher scores on the scale are directly proportional to a more impaired body image. It is designed to be applicable to patients with visceral or skin cancer and/or those undergoing cancer treatment. However, there are very few studies in the literature focusing on patients with skin cancer.^[11]^ The SCQOLIT is a 10-item scale specifically designed to assess quality of life over a 7-day period in patients with MM and NMSC, setting it apart from broader dermatological quality-of-life measures. SCQOLIT is a Likert-type questionnaire with no defined cutoff value or categorical scoring. Higher scores on the scale indicate a greater negative impact on quality of life.^[5]^

The face reflects our emotions and thoughts, playing an essential role in psychosocial life and attractiveness in social and sexual contexts. Studies have even shown that facial features can influence legal decisions.^[17]^ Lesions on the head and neck can impact quality of life, but DLQI scores suggest that our head and neck BCC patients were less affected than expected. This may be attributed to the patients being middle-aged, dealing with various other illnesses or comorbidities, perceiving the lesions as a cosmetic issue, and/or, as they age and experience other changes in their facial appearance, perceiving these lesions as ordinary and normal.

A study investigating the quality of life in patients with facial paralysis found a significant impact and decrease in their quality of life. The inability of these patients to express their emotions and thoughts was shown to negatively affect their quality of life, while an improvement in quality of life was reported following restorative treatments or interventions.^[18]^ Our study, however, investigates a different condition from facial paralysis, as BCC lesions do not affect facial expressions. The absence of deterioration in the quality of life in our patients may be attributed to their unaffected facial expressions. It is possible to conclude that, for these patients, expressions and emotions hold greater significance than visual appearance. Based on the results of this study, the lack of impact on quality of life could be attributed to this factor.

Quality of life has been largely studied in dermatological conditions such as seborrheic dermatitis, alopecia areata, rosacea, acne, and psoriasis.^[19–21]^ Facial involvement in these diseases is consistently associated with greater quality-of-life decline. However, studies specifically focusing on BCC are limited. One study reported that 89.7% of BCC patients had DLQI scores below 5, with higher scores linked to long-term sunscreen use, tumor size > 2 cm, and ulceration, but not to demographic or behavioral factors.^[22,23]^ Similarly, our study found a mean DLQI score of 1.74 ± 1.01, supporting a minimal impact on dermatological quality of life. No significant differences were observed across age, gender, education, occupation, skin type, sunscreen use, or alcohol consumption. These findings may support the idea that, unlike other inflammatory dermatoses, BCC may have a relatively limited psychosocial burden, even when lesions are located on the face. No statistically significant differences were found in the DLQI based on BCC type, localization, or diameter. This may be attributed to the fact that BCC typically affects more localized areas compared to the dermatoses mentioned above, thus having less widespread involvement of the face. Additionally, in age-related dermatoses such as acne, quality of life may be influenced by other factors. Therefore, further investigation addressing these aspects is recommended.

Unlike inflammatory dermatoses, BCC is generally a solitary lesion and does not cause widespread skin involvement. The low DLQI scores observed in our study may reflect this localized presentation and the lack of involvement of functional areas such as the hands and/or genital region. In contrast, chronic conditions like acne, psoriasis, or atopic dermatitis often affect larger surface areas and younger populations may lead to greater psychosocial burden due to these facts.^[24–28]^ These differences may partly explain the relatively preserved quality of life observed in our BCC cohort. Interestingly, DLQI scores were higher in nonsmokers in our study. Although unexpected, this may be influenced by unmeasured variables such as coping mechanisms or social factors, and warrants further investigation. A previous study using the Skindex scale reported better quality of life in BCC patients with smaller lesions and non-visible tumor locations.^[29,30]^ In contrast, our study found no significant quality-of-life impairment, even in patients with head and neck BCC. These differences may be related to sample size, cultural perceptions of appearance, or patients viewing their lesions as cosmetic problems rather than medical concerns. Additionally, the heterogeneous educational levels in our cohort may have influenced perceptions of disease severity.

A study found a strong link between psychosocial factors and quality of life in MM patients. According to the Chronic Disease and Disability Model, quality of life is a fundamental negative outcome of chronic illness. The adaptation process to the disease involves responses to the illness and clinical variables, as well as sociodemographic characteristics, psychosocial factors, and contextual factors that affect quality of life. The model also predicts mediating relationships between contextual variables and quality of life.^[31]^ The absence of these parameters and the early intervention in BCC, preventing chronicity, may explain the lack of impact on quality of life observed in our study.

The SCQOLIT is a disease-specific tool developed to evaluate the quality of life in patients with MM and NMSC, unlike less specific DLQI. Despite the global rise in skin cancer incidence, studies using SCQOLIT in NMSC remain limited. In SCQOLIT, higher scores indicate poorer quality of life. The original validation study reported a mean score of 4 in NMSC patients.^[5]^ Karakok et al later found a higher mean score of 9.6, both suggesting that NMSC, covering BCC, may have limited impact on quality of life.^[6]^ In our study, the mean SCQOLIT score was 5.56. No significant associations were found between SCQOLIT scores and variables such as gender, age, smoking or alcohol use, sunscreen habits, Fitzpatrick skin type, education, BCC subtype, tumor size, or location. While Karakok et al reported age-related differences, our study included only patients aged 45 to 65 to minimize cognitive variability. Further research should explore quality of life in elderly populations specifically. In this study, no statistically significant results were found between the DLQI and SCQOLIT scales. While this study does not determine whether SCQOLIT can replace DLQI for assessing the quality of life in skin cancer patients, broader use of SCQOLIT in future research may provide a clearer perspective and potentially change this view.

Body image, shaped by internal and external factors, is a dynamic concept affecting individuals’ thoughts, emotions, behaviors, and overall quality of life. It plays a role in self-perception, communication, disease progression, social functioning, and treatment decisions and adherence.^[22]^ The BIS was developed by Hopwood et al in 2001, consisting of 10-item self-report tool originally validated in cancer populations.^[11]^ It has been applied in breast, head and neck, colorectal cancers, and benign gynecologic conditions. Although body image has been studied in skin malignancies using various instruments, no prior study has evaluated it with the BIS in patients having skin cancer, including BCC. BIS is notable for its brevity and ease of use but remains underutilized in dermatologic oncology. Our study is the first to assess BIS specifically in BCC patients. A BIS score ≥10 has been associated with increased risk of depression and anxiety, suggesting the importance of regular screening for psychological distress.^[32]^ In our cohort, scores ranged from 1 to 7, with a mean of 3.16 ± 1.27, all below the clinical threshold. This suggests that BCC has a limited impact on body image, potentially due to its relatively lower psychological burden and its perception as less threatening compared to “real” internal malignancies. In our study, these parameters were not assessed but remained as predictions. Body image reflects an individual’s attitudes, perceptions, and behaviors related to their appearance and is often linked to psychological issues such as depression, anxiety, eating disorders, and low self-esteem.^[33]^ In our study, the possibility of psychiatric disorders influencing body image was eliminated due to the exclusion criteria for these conditions, and no impact on body image was observed. This constitutes a strong point of our study.

Body image has been more extensively studied in breast cancer, most probably due to greater public awareness and the emphasis on routine self-examinations. In contrast, awareness of skin cancer and its psychosocial consequences still remains limited.^[34]^ Body image appears to be more affected in cancers where awareness is higher and issues such as sexual function are more prominent, as in breast cancer.^[35]^ In comparison, BCC is often perceived as a minor condition or a natural consequence of skin aging, and may be dismissed as a harmless lesion such as a sunspot, particularly among older individuals. This perception, along with the advanced age of our cohort, may explain the minimal impact on body image observed in our study. Further comprehensive evaluations are needed to explore these contributing factors in depth.

In this study, a statistically significant difference was found between alcohol use and BIS scores (P = .014), with alcohol users having higher BIS scores. Given that an increase in BIS score suggests a worsening body image, body image disturbance was more frequently observed in alcohol users with BCC. This suggests that alcohol use, when combined with the disease, may impair body perception. However, since alcohol alone can also affect body perception, comparative studies will be helpful for clarification. No statistically significant differences were found between body image and factors such as age, gender, skin type, education level, occupation, BCC type, BCC localization, tumor size and number, age at first appearance of BCC, lesion duration, or smoking status in our study.

Maciel et al reported that patients with face and neck localized skin cancers exhibited high psychological morbidity due to the potential skin deformities caused by the tumor.^[36]^ Additionally, it has been reported that changes in body image can have a significant emotional, behavioral, and cognitive impact on patients, leading to considerable pain and shame. Although our study evaluated head and neck-localized BCCs, no significant differences in body image were observed. However, cognitive and emotional functions were not assessed in our study, and psychometric measurements were not conducted. It is anticipated that the results might differ if these parameters were evaluated in conjunction with the other factors.

Our study found no connection between tumor localization and gender at diagnosis, which differs from the findings in the literature. Additionally, our study did not assess scales after treatment, which is a limitation. Zivković et al found that patients with longer tumor duration reported greater concerns, symptoms, negative emotions, and lower quality of life.^[37]^ In our study, no significant correlation was detected between SCQOLIT, BIS, DLQI scores, and tumor age, contrary to the existing literature.

In this study, we conducted inferential statistical analyses to explore whether quality of life (DLQI, SCQOLIT) and body image (BIS) scores varied by sex, age group, tumor location, and lesion size. However, no statistically significant differences were found for any of these variables. Notably, a weak negative correlation was observed between the number of BCC lesions and SCQOLIT scores (*R* = −0.222, P < .05), suggesting that having multiple lesions may slightly reduce the perceived quality of life (these findings are detailed in Tables 5–9). Based on all the assessments mentioned above, it is clear that there may be an interaction between quality of life and body image. In our study, no statistically significant relationship was found between DLQI and BIS (P > .05). However, a positive moderate correlation was found between SCQOLIT and BIS (*R* = 0.300). In other words, as the score of one scale increases, the other also shows a positive increase. This indicates that as body image worsens in BCC patients, skin cancer-related quality of life declines, although DLQI remains unaffected. This study highlights that SCQOLIT may be a more suitable scale than DLQI for evaluating body image perception and quality of life in NMSC patients.

The prospective nature of our study, along with the absence of time constraints in BIS, allowing for a more comprehensive reflection of the general population, are key strengths of this study. Additionally, while many quality-of-life indices related to skin cancers have been defined, SCQOLIT is the only one specifically adapted for nonmetastatic skin cancers, and its limited use in the literature makes its application in our study a distinguishing factor.

Our study has some limitations. The relatively small sample size does not fully reflect the general population. Additionally, the lack of posttreatment DLQI, BIS, and SCQOLIT data, as well as the fact that SCQOLIT is based on the past week and does not provide information about cosmetic concerns, are limitations of the study. Furthermore, parameters such as social support, which could influence quality of life and body image in individuals with skin tumors, were not included in this study. Another limitation of this study is the lack of reassessment of these parameters following BCC treatment, as well as the absence of an evaluation of the potentially beneficial effects of social support.

Although excluding patients over 65 years old and those with psychiatric conditions may reduce the generalizability of our results, these criteria were chosen consciously. We aimed to protect the internal validity of the study by minimizing factors such as cognitive decline or psychiatric symptoms that might interfere with the self-reported scales. Since the design of this study was observational and we could not conduct formal cognitive or psychiatric evaluations, we preferred to include a more stable population, even if it meant having fewer patients. Future studies including neurologic or psychiatric evaluations could help extend the findings to larger populations.

In this study, the low DLQI and SCQOLIT scores suggest that BCC has a limited impact on patients’ overall quality of life, even in visible head and neck localizations. Interestingly, nonsmoker patients reported significantly higher DLQI scores, though the clinical relevance of this finding remains unclear. No significant correlation was observed between DLQI and SCQOLIT, indicating that these scales may reflect different aspects of patient experience and cannot be used interchangeably. However, a moderate positive correlation was found between SCQOLIT and BIS scores, suggesting that as body image deteriorates, quality of life related to skin cancer also declines. This implies that SCQOLIT may be more sensitive than DLQI in capturing psychosocial dimensions such as body image in patients with BCC.

As the first study to compare these 3 instruments in this context, our findings highlight the potential value of incorporating SCQOLIT into future research and clinical assessments for NMSCs including BCC.

## Author contributions

**Conceptualization:** Nazli Caf.

**Data curation:** Ozan Yildirim.

**Formal analysis:** Nazli Caf, Mustafa Tümtürk.

**Methodology:** Ozan Yildirim, Nazli Caf.

**Project administration:** Ozan Yildirim, Nazli Caf, Zafer Türkoğlu.

**Software:** Mustafa Tümtürk.

**Supervision:** Nazli Caf, Zafer Türkoğlu.

**Validation:** Ozan Yildirim, Nazli Caf.

**Writing – original draft:** Ozan Yildirim, Nazli Caf, Mustafa Tümtürk.

**Writing – review & editing:** Ozan Yildirim, Nazli Caf, Zafer Türkoğlu.
